# Factors Associated With Nurses’ Acceptance of Videoconferencing in Telenursing: An Integrated TAM–TPB Analysis of Behavioral Determinants and Prior Training

**DOI:** 10.1111/inr.70263

**Published:** 2026-11-14

**Authors:** Liora Shmueli, Svetlana Kats

**Affiliations:** ^1^ Department of Management Bar‐Ilan University Ramat‐Gan Israel; ^2^ Department of Health Policy Stanford University School of Medicine Stanford USA

**Keywords:** nurses, technology acceptance model, telehealth, telenursing, theory of planned behavior, training, videoconferencing

## Abstract

**Aim:**

To examine nurses' willingness to adopt videoconferencing for telenursing using an integrated technology acceptance model and theory of planned behavior framework, with a specific focus on the role of prior telenursing training.

**Background:**

Successful telenursing implementation depends on behavioral factors and the association between prior training and nurses’ acceptance of remote communication tools. While technical feasibility is often studied, the social and psychological drivers of adoption remain under‐explored in nursing policy.

**Methods:**

A cross‐sectional online survey of 330 registered nurses was conducted (November 2022–January 2023). Participants were recruited via professional social media groups. Data were analyzed using hierarchical multivariate regression based on an integrated behavioral framework.

**Results:**

The integrated framework explained 74% of the variance in intention to use telenursing. In the final multivariable model, perceived usefulness, attitudes toward use, subjective norms, and perceived behavioral control were independently associated with intention to use telenursing, whereas perceived ease of use and self‐efficacy were not. Nurses with prior telenursing training reported higher willingness to use videoconferencing and more positive subjective norms and perceived behavioral control in the unadjusted analyses. However, prior training was not independently associated with intention after adjustment.

**Discussion:**

While utilitarian drivers like perceived usefulness are important, social and environmental factors are critical. Utility alone is insufficient to drive adoption; nurses' intentions are deeply embedded in the social and organizational structures of clinical settings.

**Conclusions:**

The integrated TAM–TPB model offers a comprehensive understanding of nurses' acceptance of videoconferencing in telenursing. Although prior telenursing training was not independently associated with intention after adjustment for the TAM and TPB constructs, it was associated with more positive social perceptions and perceived behavioral control in the unadjusted analyses. Further longitudinal and interventional studies are needed to determine whether structured telenursing training contributes to the implementation and adoption of telenursing in clinical practice.

**Implications for Nursing:**

Competency‐based telenursing training and supportive leadership may support nurses' readiness for telenursing; however, the effectiveness of such interventions should be evaluated in future longitudinal and interventional studies.

**Implications for Health Policy:**

The findings suggest that health systems and policymakers may consider strategies to strengthen social support and organizational readiness for telenursing, such as peer mentorship programs and protected administrative time for remote care.

## 1 Introduction

Telenursing is a form of telehealth that uses technology to deliver nursing care remotely. By leveraging telecommunication technologies, telenursing facilitates the management and coordination of healthcare services across geographic boundaries. Its applications span a wide spectrum, encompassing remote patient monitoring, live video consultations, and virtual triage assessments (Glinkowski et al. 2013; Pereira Sousa et al. 2022).

Historically, telenursing dates back to the introduction of the telephone, which enabled nurses to address healthcare inquiries remotely. Over the years, telenursing has undergone a transformative evolution beyond mere phone triage to include videoconferencing as a vital component of its services (Chan and Hjelm 2001; Rutledge and Gustin 2021). The COVID‐19 pandemic catalyzed this digital revolution in healthcare due to the necessity to change the work environment of professionals, particularly nurses. The outcome has been a global acceptance of digital technologies and virtual platforms, such as telehealth and telenursing, and, consequently, the redefinition of the roles and responsibilities of nurses within the healthcare industry (Bell and Brysiewicz 2020).

To meet these changing roles, nurses engaging in telenursing need to possess a range of skills and competencies across various dimensions, including professional, methodological, personal, and social aspects. Key competencies include strong communication skills, the ability to compensate for the lack of visual assessment, deep clinical knowledge, and the ability to apply nursing expertise in a telehealth setting (van Houwelingen et al. 2016). For instance, care delivery through audio calls requires greater interviewing competency, as nurses lose the possibility of using visual assessment for decision‐making. While many of these skills are fundamental nursing practices, their specific application to the telehealth context requires additional training within nursing education and healthcare organizations to prepare nurses for effective telenursing practices (Fincher et al. 2009).

Beyond efficiency, telenursing videoconferencing offers numerous benefits, including facilitating person‐centered personalized nursing, fostering self‐management, and encouraging patients and families to actively participate in their healthcare. This approach holds particular value for individuals residing in rural or remote regions, as it effectively mitigates distance‐related barriers, thereby enhancing access to essential healthcare services (Gajarawala and Pelkowski 2021).

However, despite these advantages, integrating videoconferencing presents personal, organizational, and professional challenges for nurses. These challenges include the necessity for adequate training and support, as well as the potential implications for professional practice. In light of the increasing popularity of telenursing, it is essential to comprehend the factors associated with nursing professionals' acceptance of the practice to successfully implement and maintain its integration into healthcare systems. This is doubly important given that nurses' perceptions directly impact patient satisfaction, clinical outcomes, and performance (Ajzen 2020; Taherdoost 2019).

Despite the rapid expansion of telenursing, empirical evidence examining the behavioral determinants of nurses’ acceptance of videoconferencing‐based care remains limited. Two widely used frameworks to evaluate factors associated with technology acceptability are the theory of planned behavior (TPB) and the technology acceptance model (TAM). Perceived usefulness (PU), perceived ease of use (PEOU), and attitude toward usage are the factors the TAM uses to determine acceptance (Venkatesh et al. 2003). The TPB model considers a person's acceptance of technology to be dependent on several factors, such as (1) the perceived social pressure from significant others to engage in the behavior (subjective norms); (2) the person's perceived ease or difficulty in performing the behavior (perceived behavioral control, PBC) (Venkatesh et al. 2003); and (3) the belief in one's ability to overcome potential obstacles (self‐efficacy) (Ajzen 2020).

Only a few studies to date have explored the integration of both TAM and TPB in the context of telehealth; moreover, evidence from a recent systematic review indicates that research on healthcare professionals’ acceptance of remote care technologies has largely relied on standalone or modified TAM‐ or UTAUT‐based frameworks and has examined telehealth in generic terms, leaving the integrated TAM–TPB models in telenursing, especially videoconferencing‐based nursing care, largely unexplored (Rouidi et al. 2022).

One such study sought to integrate the TPB, TAM, and self‐determination theory in order to explore the adoption behavior of a telehealth system, focusing more on reporting the path coefficients in the proposed model (Hsieh et al. 2022). Another study, applying the decomposed TPB (DTPB) as a research framework, found that facilitating conditions and supervisor influence were associated with nurses' behavioral intentions, and together these variables accounted for 28.6% of the variance (Chang et al. 2021).

To address these gaps, this study utilizes an integrated TAM–TPB framework to evaluate whether a unified model provides greater explanatory value for nurses' intention to adopt videoconferencing compared to standalone models (see Supplementary Figure S1). The study cohort encompassed a wide range of nurses working in different settings.

## 2 Materials and Methods

### 2.1 Study Design and Participants

This study employed a cross‐sectional online survey design. Participants were recruited using a convenience sampling approach between November 29, 2022, and January 3, 2023, through a survey distributed across various professional Facebook groups targeting registered nurses working in diverse healthcare settings in Israel. Eligible participants were registered nurses currently employed in the Israeli healthcare system, aged 18 years or older. Registered‐nurse status was self‐reported through an eligibility screening question at the beginning of the questionnaire (*“Are you currently employed as a nurse in the healthcare system?”*). Only participants answering “Yes” were eligible to continue the survey. Professional registration was not independently verified. A total of 561 individuals accessed the survey. Of these, 231 did not complete the questionnaire and were excluded. The final analytical sample comprised 330 registered nurses. Only completed questionnaires were included in the final analysis. Incomplete questionnaires were excluded. Responses were reviewed for potential duplicate submissions, and no duplicate responses were identified.

Facebook was selected as the recruitment platform to facilitate access to a geographically dispersed and broad population of nursing professionals across various clinical specialties and organizational tiers. Furthermore, this method provided a convenient and accessible point of entry for participants, allowing them to engage with the survey in a familiar digital environment.

Participants were recruited through convenience sampling, with a target of at least 300 nurses in total. As per established statistical practices, we used 10–15 participants per predictor variable, which is appropriate for regression models involving six or more predictors, as it ensures a good balance between the number of participants and the number of variables in the study (Wilson Van Voorhis and Morgan 2007).

### 2.2 Ethical Considerations and Consent to Participate

The study was approved by the Ethics Committee for Nonclinical Studies, Bar‐Ilan University, Israel, on November 17, 2022. All procedures were performed in accordance with the ethical principles of the Declaration of Helsinki for research involving human participants.

The questionnaire was conducted using Qualtrics software. In the software, data are stored without containing any personal information that could compromise participants' privacy. Informed consent for participating in the study was obtained digitally through Qualtrics software from all subjects, and all methods were carried out following the relevant guidelines and regulations of the Ethics Committee for Nonclinical Studies at Bar‐Ilan University, Israel. Specifically, at the beginning of the questionnaire form, the participants were informed that their participation was voluntary and that they were permitted to terminate their participation at any time and were requested to confirm their informed consent to participate in the research.

The participants received a full explanation of the structure and nature of the study and that it was anonymous. Participants who refused to give their consent to proceed with the questionnaire and those under the age of 18 years were excluded. No incentives were provided to the study participants.

### 2.3 Questionnaire

The following sections describe the dependent and independent variables in the questionnaire and their operationalization in this study. The questionnaire consisted of TAM, TPB (the items appear in Table 1), accessibility to patients, previous telenursing training and sociodemographic characteristics. The parameters comprising the study measurements used to build the conceptual model and hypotheses are described in Supplementary Figure S1.

**TABLE 1:** Items, response scales and internal consistency for assessing measures of two models: TAM and TPB.

Model/Theory	Measures	Items	Mean	Std	α
TAM	Attitude toward use (AT)	Using telenursing technology in patient care and management is a good idea.	4.31	1.03	.90
		Using telenursing technology in patient care and management is pleasant.			
		Using telenursing technology is beneficial to my patient care and management.			
		Using telenursing technology facilitates better patient care decision‐making.			
		^*^Using telenursing technology represents a violation of patient privacy.			
		^*^Using telenursing technology causes nurses to give less time to quality patient care.			
TAM	Perceived usefulness	Using telenursing can enable me to complete patient care more quickly.	4.09	.99	.92
		Using telenursing can improve my patient care and management.			
		Using telenursing can increase my productivity in patient care.			
		Using telenursing can enhance my service effectiveness.			
		Using telenursing can make my patient care and management easier.			
		I would find telenursing technology useful for my patient care and management.			
TAM	Perceived ease of use	Learning to operate telenursing technology would be easy for me.	4.54	.94	.93
		I would find it easy to get telenursing technology to do what I need to do in my patient care and management.			
		My interactions with telenursing technology would be clear and understandable.			
		I find telenursing technology flexible to interact with.			
		It is easy for me to become skillful in using telenursing technology.			
		I would find telenursing technology easy to use.			
TAM	Intention to use (ITU)	I intend to use telenursing technology in my patient care and management when it becomes available in my department or hospital.	4.17	1.19	.96
		I intend to use telenursing technology to provide health care and management routinely.			
		Whenever possible, I intend to use telenursing technology in my patient care and management.			
		To the extent possible, I would use telenursing to do different things, clinical or nonclinical.			
		To the extent possible, I would use telenursing in my patient care and management frequently.			
TPB	Subjective norms	Colleagues at my health care services think that I should use telenursing technology.	3.73	1.07	.81
		My supervisors, who are assessing my patient care and management, think that I should use telenursing technology.			
TPB	Perceived behavioral control	I would have the ability to use telenursing technology in my patient care and management.	4.18	1.05	.87
		I would have the knowledge to make use of telenursing technology in my patient care and management.			
		I would have the resources to make use of telenursing technology in my patient care and management.			
TPB	Self‐efficacy	I could complete a job or a task using telenursing if I could call someone for help if I get stuck.	4.52	1.04	.78
		I could complete tasks in my work using the telenursing system if I receive the relevant training.			
	Accessibility to patient	Telenursing technology will allow me to treat patients who live at further distances.	4.92	1.03	.93
		Telenursing technology will allow me to be in contact with patients who seldom come to the clinic.			
		Telenursing technology will allow me to be in contact with patients who cannot be easily brought to the clinic.			

Items Response scale: 1–6 agreement.

* Negative items were reverse scored.

### 2.4 Measurement and Variables

The dependent variable was the intention to use telenursing technology via videoconferencing, determined by five items measured on a 1–6 scale (1‐strongly disagree, 6‐strongly agree).

The independent variables were: (1) Sociodemographic characteristics: (a) age; (b) gender; (c) education; and (d) seniority; (2) TAM covariates were measured by six items for each covariate: perceived usefulness, PEOU, and attitude. (3) TPB covariates: subjective norms, measured by two items: PBC, measured by three items, and self‐efficacy, measured by two items. (4) Accessibility to patients, measured by three items. Accessibility to patients refers to the degree to which the nurse believes the use of the telenursing service will increase contact with underserved patients who live in regions that are geographically distant from medical facilities or who have difficulty getting to a medical facility.

All the TAM, TPB, and accessibility to patient items were measured on a 1–6 scale (1, strongly disagree; 6, strongly agree). Negative items were reverse scored, so that higher scores indicated higher levels of the item. Scores for each item were averaged to obtain each of the TAM and TPB independent categories.

#### 2.4.1 Reliability of the Questionnaire

A Cronbach's α internal reliability method revealed that the internal consistency of TAM was Cronbach's α = .96, and that of TPB was Cronbach's α = .90. The internal consistency of the integrated model was Cronbach's α = .97 (Table 1). The Cronbach's α values demonstrate strong internal consistency for our measures, indicating the reliable measurement of intended constructs and enhancing the questionnaire's validity.

### 2.5 Statistical Analysis

Data processing and analysis were done using SPSS for Windows software (Version 28) and the Process add‐on for SPSS (Version 3.5). Descriptive statistics of the independent variables and demographic characteristics were calculated for the overall population of nurses. Interactions between dependent and independent discrete variables were examined using reliability tests, according to the specific characteristics of the variables, using standard reliability tests (*t*‐test and chi‐square test, according to the specific characteristics of the variables).

To assess the construct validity of the measurement instruments, confirmatory factor analysis (CFA) was conducted using AMOS (Version 28) to evaluate the hypothesized eight‐factor structure. Model fit was assessed using multiple fit indices. Overall, the results supported the proposed measurement model, with most indices indicating adequate fit. The CFA results indicated adequate model fit: $\chi^{2}\left(406\right)=791.24,p<.001$. RMSEA = .05, CFI = .93, and TLI = .92. Detailed CFA results and standardized factor loadings are presented in Supplementary Figure S2.

Multicollinearity was assessed using the variance inflation factor (VIF), and no missing data were present in the data set; therefore, no imputation procedures were required.

Bivariate analyses were then performed to examine correlations between factors that might be associated with the dependent variable (intention to use telenursing) and the independent variables (i.e., sociodemographic factors and variables from the TAM, TPB, accessibility to patients, and telenursing past training). Finally, we performed a multivariate analysis to investigate determinants of intention to use telenursing by using a multivariate ordinary least squares (OLS) regression analysis, which enabled us to examine the distinct independent associations of each independent variable on the dependent variable. Influential observations were assessed using Cook's distance. All Cook's distance values were below 1, indicating that no influential observations were identified. As a sensitivity analysis, an additional four‐step hierarchical regression model was conducted in which gender, education, and seniority were entered in the first step, followed by the TAM constructs, TPB constructs, and finally accessibility to patients and prior telenursing training. The complete results are presented in Supplementary Table S2.

## 3 Results

### 3.1 Participant Characteristics and Univariate Analysis

Overall, 330 participants completed the online survey. Of the participants, 22.4% (*n* = 74) were male, and 77.6% (*n* = 256) were female. The average age of the participants was 39.28 (SD = 8.5), and the average seniority was 11.39 years (SD = 8.25). The majority of the participants, 61.2% (*n* = 202), had a BA academic degree, and 38.8% (*n* = 128) had an MA or higher academic degree. Most of the participants did not receive telenursing training in the past (89.7%). See Table 2 for full results.

**TABLE 2:** Sociodemographic characteristics of the research sample (*n* = 330).

		(*n* = 330)			
Variable	Subgroup	M	SD	*N*	%
Age		39.28	8.50		
Seniority		11.39	8.25		
Gender					
	Male			74	22.40%
	Female			256	77.60%
Education					
	BA			202	61.20%
	MA or higher			128	38.80%
Telenursing past training					
	No			296	89.70%
	Yes			34	10.30%

#### 3.1.1 Bivariate Correlations

To test the bivariate correlations of all the TAM variables, TPB variables, accessibility to patients, and the two continuous demographic variables (age and seniority) with the intention to use telenursing, all pairwise Pearson correlation coefficients were calculated. The mean level of intention to use telenursing reported by the nurses was 4.17 (SD = 1.19) and was positively and significantly correlated with all TAM variables (i.e., attitude toward use (*r* = .75, *p* < 01), perceived usefulness (*r* = .74, *p* < 0.01), and PEOU (*r* = .59, *p <* 0.01)), all TPB variables (i.e., subjective norms (*r* = .70, *p* < 0.01), PBC (*r* = .74, *p*. < 0.01), and self‐efficacy (*r* = .57, *p* < 0.01)) and accessibility to patients (*r* = .63, *p* < 0.01). However, the study did not find any correlation between age or seniority and the intention to use telenursing. See Table 3 for full results.

**TABLE 3:** Pearson correlations.

Variable	M	SD	1.	2.	3.	4.	5.	6.	7.	8.	9.
1. Intention to use telenursing	4.17	1.19									
2. Attitude toward use	4.31	1.03	.746^**^								
3. Perceived usefulness	4.09	.99	.740^**^	.787^**^							
4. Perceived ease of use	4.54	.94	.594^**^	.530^**^	.549^**^						
5. Subjective norms	3.73	1.07	.698^**^	.574^**^	.591^**^	.425^**^					
6. Perceived behavioral control	4.18	1.05	.738^**^	.579^**^	.577^**^	.653^**^	.682^**^				
7. Self‐efficacy	4.52	1.04	.570^**^	.508^**^	.489^**^	.452^**^	.553^**^	.644^**^			
8. Accessibility to patient	4.92	1.03	.625^**^	.605^**^	.611^**^	.569^**^	.533^**^	.634^**^	.678^**^		
9. Age	39.28	8.50	.037	.071	.002	–.061	.050	<.001	.001	.017	
10. Seniority	11.39	8.25	.099	.127^*^	.070	–.048	.117^*^	.063	.047	.002	.747^**^

*Note*: *p < .05, **p < .01 (2‐tailed).

#### 3.1.2 Univariate Analysis

To test differences in the intention to use telenursing between men and women, an independent samples *t*‐test was conducted. The results of the test indicated no significant difference between the sexes (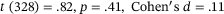). Specifically, no difference in the intention to use telenursing was found between men (M = 4.07, SD = 1.20, *n* = 74) and women (M = 4.20, SD = 1.19, *n* = 256).

An independent samples *t*‐test of the differences in the intention to use telenursing between nurses of different education levels (BA vs. MA or higher) indicated no significant difference ($t\;\left(328\right)=.96,p=.34,\;{\mathrm{Cohen}}^{\prime }s\;d=.11$). Specifically, the intention to use telenursing was similar among nurses with a BA degree (M = 4.12, SD = 1.22, *n* = 202) and those with an MA degree or higher (M = 4.25, SD = 1.14, *n* = 128).

In contrast, the factor of prior training on telenursing was associated with higher intention to use telenursing, with an independent samples *t*‐test indicating a significant difference (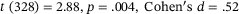). Specifically, those with past training (M = 4.72, SD = 1.03, *n* = 34) reported higher intention to use telenursing than those without past training (M = 4.10, SD = 1.19, *n* = 296).

We also conducted univariate tests of the differences in all TAM and TPB variables between nurses with prior training in telenursing and those with no prior training.

Our results indicate that prior training was associated with substantially stronger subjective norms $t=3.73p<.001,\;{\mathrm{Cohen}}^{\prime }s\;d=.68,\;\mathrm{medium}\;\mathrm{effect}$), and higher PBC $t=3.53p<.001,\;{\mathrm{Cohen}}^{\prime }s\;d=.64,\;\mathrm{medium}\;\mathrm{effect}$), indicating that prior training was associated with more favorable subjective norms and PBC.

In contrast, no significant differences were observed between the groups with respect to attitudes toward use ($t=1.87p=.06,\;{\mathrm{Cohen}}^{\prime }s\;d=.34,\;\mathrm{small}\;\mathrm{effect}$), perceived usefulness (*t* = 1.02, *p* = .31, ${\mathrm{Cohen}}^{\prime }s\;d$ = 0.18, smalleffect), or PEOU (*t* = 1.03, *p* = .31, ${\mathrm{Cohen}}^{\prime }s\;d$ = 0.19, smalleffect) (see Supplementary Table S1).

### 3.2 Multivariate Analysis

A hierarchical multiple OLS regression analysis was conducted in three steps to examine predictors of intention to use telenursing via videoconferencing. In Step 1, core constructs of the TAM were entered. In Step 2, TPB variables were added to assess their incremental explanatory value beyond TAM. In Step 3, prior telenursing training and accessibility to patients were added.

The initial TAM model accounted for 65% of the variance in the intention to use telenursing (*R* ^2^ = .65, F(3, 326) = 201.69, *p* < .001 (Table 4, Model 1). The addition of TPB model in Step 2 significantly improved the model (ΔR^2^ = .09, ΔF (3,323) = 38.90, *p* < .001), bringing the total explained variance to 74% (F (6,323) = 155.46, *p* < .001, *R* ^2^ = .74, adjusted *R* ^2^ = .74). Adding training and accessibility in Step 3 did not improve the model (ΔR^2^ = .001, ΔF(2, 321) = 0.42, *p* = .66; final model F(8, 321) = 116.28, *p* < .001, *R* ^2^ = .74).

**TABLE 4:** Multivariate analyses of intention to use telenursing.

Predictor variable	Model 1: TAM						Model 2: TAM + TPB						Model 3:TAM + TPB + Training + Accessibility					
	B (SE)	β	95% CI for B	t	p	VIF	B (SE)	β	95% CI for B	t	p	VIF	B (SE)	β	95% CI for B	t	p	VIF
**TAM factors**																		
Attitude toward use	0.43 (0.06)	0.37	[0.31, 0.56]	6.92	<.001	2.72	0.30 (0.06)	0.26	[0.19, 0.41]	5.44	<.001	2.89	0.30 (0.06)	0.26	[0.19, 0.41]	5.29	<.001	2.94
Perceived usefulness	0.39 (0.07)	0.33	[0.26, 0.52]	5.97	<.001	2.80	0.26 (0.06)	0.22	[0.14, 0.37]	4.44	<.001	2.98	0.26 (0.06)	0.22	[0.14, 0.38]	4.37	<.001	3.08
Perceived ease of use	0.27 (0.05)	0.22	[0.17, 0.37]	5.41	<.001	1.48	0.09 (0.05)	0.07	[−0.01, 0.18]	1.71	.09	1.95	0.08 (0.05)	0.07	[−0.02, 0.18]	1.64	.10	2.02
**TPB factors**																		
Subjective norms	—	—	—	—	—	—	0.22 (0.05)	0.20	[0.12, 0.31]	4.64	<.001	2.22	0.21 (0.05)	0.19	[0.12, 0.30]	4.48	<.001	2.26
Perceived behavioral control	—	—	—	—	—	—	0.31 (0.06)	0.27	[0.20, 0.42]	5.55	<.001	3.06	0.30 (0.06)	0.27	[0.19, 0.42]	5.29	<.001	3.16
Self‐efficacy	—	—	—	—	—	—	0.02 (0.04)	0.02	[−0.07, 0.11]	0.43	.67	1.84	0.01 (0.05)	0.01	[−0.08, 0.11]	0.30	.76	2.24
Telenursing past training	—	—	—	—	—	—	—	—	—	—	—	—	0.09 (0.11)	0.02	[−0.13, 0.32]	0.80	.42	1.09
Accessibility to patient	—	—	—	—	—	—	—	—	—	—	—	—	0.02 (0.05)	0.02	[−0.08, 0.13]	0.44	.66	2.53

Model Statistics				
Model	R^2^	ΔR^2^	F	ΔF
Model 1	.65	—	F(3,326) = 201.69, *p* <.001	—
Model 2	.74	.09	F(6,323) = 155.46, *p* <.001	ΔF(3,323) = 38.90, *p* <.001
Model 3	.74	.001	F(8,321) = 116.28, *p* <.001	ΔF(2,321) = 0.42, *p* = .66

*Note*: B = unstandardized regression coefficient; SE = standard error; β = standardized regression coefficient; 95% confidence intervals are reported for the unstandardized regression coefficients (B); VIF = variance inflation factor.

An examination of the model coefficients indicated that attitude toward use (β=.26,p<.001), perceived usefulness (β=.22,p<.001), subjective norms (β=.2,p<.001), and PBC (β=.27,p<.001) were positively associated with intention to use telenursing via videoconferencing. Notably, perceived ease of use (β = .07, p = .09) and self‐efficacy (β = .02, p = .67) were not significantly associated with intention.

In the final multivariate integrated model, PEOU shifted from a significant predictor (β = .22, *p <* .001) to nonsignificance (β = .07, *p* = .1). Similarly, self‐efficacy did not emerge as a significant predictor (β = .01, *p* = .76). The moderate‐to‐high correlations observed between PEOU, self‐efficacy, and PBC further suggest that these constructs share overlapping variance. This pattern may indicate that for this sample of registered nurses, broader perceptions of control over technology use, including organizational support and readiness for technology integration, overlap with PEOU and self‐efficacy in explaining behavioral intention.

Accessibility to patients was significantly associated with intention to use telenursing in the bivariate analysis. However, when entered together with prior telenursing training in the final step of the hierarchical regression, it did not provide a significant incremental contribution beyond the TAM and TPB constructs (accessibility B = 0.02, 95% CI [−0.08, 0.13], *p =* .66; training B = 0.09, 95% CI [−0.13, 0.32], *p* = .42; ΔR^2^ = .001, *p* = .66).

Despite moderate‐to‐high correlations among some predictors (Table 3), multicollinearity diagnostics based on VIF values indicated acceptable levels of collinearity (all VIFs ≤ 3.16, below the conventional cutoff of 5; Table 4).

A sensitivity analysis adjusting for gender, education, and seniority yielded results consistent with the primary analysis, with no meaningful changes in the direction, magnitude, or statistical significance of the associations (Supplementary Table S2).

See Table 4 for the full model of the coefficients.

## 4 Discussion

This study emphasizes the importance of understanding nurses' perceptions of videoconferencing for telenursing by using the TAM and TPB models. By integrating these frameworks, we gained a detailed understanding of the factors that are associated with nurses' attitudes and their intention to use this technology. As telehealth and remote learning tools become more important in nursing practice and education, it is crucial to optimize the acceptance and effectiveness of technologies that enable remote communication with patients. This is especially important when it comes to nurses, who play a significant role in patient communication through telehealth.

This study aimed to examine the explanatory value of an integrated TAM–TPB framework and investigate behavioral factors associated with nurses' intention to use telenursing via videoconferencing, compared with either model alone. To improve explanatory power, we combined the two models. While TAM effectively captures factors that are associated with technology adoption, such as perceived usefulness, it often overlooks the social and environmental factors in clinical settings. By incorporating TPB's constructs of subjective norms and PBC, our unified framework accounts for the professional and social factors that shape a nurse's decision‐making process.

This TAM–TPB unified model's predictor variables explained 74% of the variance in the intention to use telenursing through videoconferencing, which was considerably higher than that obtained using each of the two behavior models separately. This finding aligns with nursing informatics literature suggesting that unified models (like TAM–TPB or UTAUT) generally explain a greater proportion of the variance in behavioral intention‐framework approaches when applied to complex clinical environments. As Strudwick (2015) emphasized in an integrative review of technology use among nurses, nurses’ intentions are not driven by utility alone; rather, they are deeply embedded in social and organizational structures.

The high percentage in the nurses' telenursing study suggests that the unified TAM–TPB model is especially well‐suited for that specific context. In contrast, the widely used TAM or TPB alone typically explains less of the variance in technology acceptance; TAM generally explains about 40%–50% of the variance in individuals' intention to use technology, and TPB shows similar results. The UTAUT model, which incorporates elements from TAM and TPB, was found to account for 70% of the variance in behavioral intention (Lampo 2023).

In our study, we found that attitudes toward the use and perceived usefulness based on TAM and subjective norms and PBC based on TPB were the factors most strongly associated with intention to use telenursing via videoconferencing, aligning with previous TAM and TPB studies among nurses that identified these constructs as central determinants of telehealth and digital technology adoption (Barzekar et al. 2019; Chang et al. 2021; Kats et al. 2023).

Perceived usefulness is a cornerstone in predicting healthcare professionals' adoption of telemedicine. Research consistently emphasizes its importance, highlighting perceived usefulness as a key factor in healthcare professionals' intention to adopt new technologies (Barzekar et al. 2019; Holden et al. 2012; Hu et al. 1999; Hung et al. 2014; Saigi‐Rubió et al. 2016). In a recent systematic review by Garavand et al. (2022), it was found that across various telemedicine platforms, providers consider perceived usefulness to be the most critical determinant of adoption (Garavand et al. 2022). This is particularly evident in critical care settings (eICU), where research shows that perceived usefulness is the strongest direct predictor of nurses’ intention to use telemedicine technology (Kowitlawakul 2011). Our results mirror these findings, suggesting that when telenursing is perceived as a tool that enhances clinical efficacy, nurses are more likely to integrate it into their practice.

Our findings are in line with recent studies that underscore the importance of subjective norms in determining nurses' intentions to implement telenursing. It has been suggested that promoting the development of telehealth services within an organization could encourage nurse engagement in telenursing through collaborative efforts in developing technological solutions (Chang et al. 2021). Additionally, support from colleagues and the organization is crucial in shaping nurses' intentions to use new technologies, which are essential for creating positive experiences of digitalization for healthcare professionals (Konttila et al. 2019). Furthermore, management support, including staff training, technical assistance, and technology system guidelines, plays a significant role in facilitating nurses' adoption of new technologies (Barzekar et al. 2019). Beyond the general literature, specific regional data among Israeli nurses working mainly at hospitals in Israel who undertook post‐basic nursing education training support the significant association of subjective norms with videoconferencing adoption (Kats and Shmueli 2023).

However, in the final integrated model, PEOU and self‐efficacy were not significant independent predictors of intention to use telenursing. Although both constructs demonstrated significant bivariate associations with intention to use telenursing, their associations with intention were attenuated after the inclusion of TPB‐related variables, particularly PBC. This pattern suggests that ease of use and individual confidence may operate indirectly through broader perceptions of control, organizational support, and readiness to integrate telenursing into routine clinical practice.

This finding may reflect the professional profile of the sample, which consisted of registered nurses already familiar with digital healthcare environments. In such settings, adoption intentions may depend less on whether the technology is perceived as technically easy to use, and more on whether nurses feel supported by their organization, capable of integrating the technology into existing workflows, and socially encouraged to use it. Clinically, these findings highlight that successful implementation strategies should extend beyond usability‐focused interventions and include organizational support mechanisms, structured training, mentorship, and workflow integration stratagies that may be worth evaluating for their ability to support PBC and normative support for telenursing adoption.

In the unadjusted analyses, nurses with prior telenursing training reported higher intention to use telenursing and stronger subjective norms and PBC. However, prior training was not independently associated with intention in the fully adjusted regression model after accounting for the TAM and TPB constructs. These findings should be interpreted with caution, as only 34 participants reported prior telenursing training and training was assessed using a single dichotomous (yes/no) measure without information on its content, duration, timing, or quality. This finding is consistent with previous research reporting positive associations between prior exposure to telenursing education or training and nurses' perceptions and intentions regarding telenursing (Khraisat et al. 2023). This notion is further supported by a recent study suggesting that the use of training and educational programs may facilitate the adoption of digital health technologies by healthcare professionals (Borges do Nascimento et al. 2023). In line with this, another study, conducted among undergraduate nursing students, found that the lack of formal instruction in the field may have contributed to students’ lack of substantial understanding of telenursing. To help nursing students acquire the skills and knowledge concerning telehealth technologies and telenursing required to provide safe and competent care using telehealth, telenursing education should be integrated into nursing curricula, as such training may contribute to normative support and PBC, which are key determinants of intention to adopt telenursing. This may be associated with more favorable nursing students' attitudes and intentions toward telenursing (Mun et al. 2024; Taylor and Fuller 2021). To ensure professional readiness, structured telenursing training must encompass technical proficiency, advanced digital communication strategies, and the legal, ethical, and organizational frameworks of remote care. Such programs should be integrated into both pre‐licensure curricula and continuous professional development through a multimodal approach, incorporating simulation and supervised clinical practice, supported by explicit competency standards and formal assessment (Rutledge et al. 2021). While simulation‐based training has proven highly feasible and satisfying for participants, challenges remain regarding its scalability for large workforces (Bouamra et al. 2021). Therefore, a hybrid model of digital and hands‐on simulation may be necessary to reach a high number of healthcare professionals effectively.

Subjective norms play an important role in nurses' intentions to practice telenursing according to the TPB. Previous studies have highlighted the importance of organizational support, including supervisors, peers, and leadership, in fostering positive attitudes toward telehealth adoption (Chang et al. 2021; Dolezel and Hewitt 2023; Kats and Shmueli 2023). Although structured telenursing training may contribute to these processes, its specific role requires further evaluation in longitudinal and interventional studies.

To operate telenursing systems, nurses must have both clinical expertise and appropriate technological skills. However, telehealth education remains insufficiently integrated into nursing and allied health curricula, and that lack of training is a key barrier to implementation; nursing programs still provide limited preparation in this area. Thus, despite the demonstrated benefits of telenursing and generally positive attitudes toward its use, a substantial gap persists in telehealth training and its systematic integration into nursing educational programs (Rettinger et al. 2024).

Our findings do not show an independent association of prior training with intention; training programs may still support readiness and warrant evaluation in longitudinal/interventional studies.

### 4.1 Study Limitations

It is important to acknowledge some limitations of this study. First, participants were recruited using a convenience sampling approach through Facebook groups, which may have introduced self‐selection bias and may limit the generalizability of the findings to the entire population of nurses. Nurses who are active on social media and willing to participate in online surveys may differ from the general nursing workforce, particularly in terms of digital literacy, attitudes toward technology, and prior exposure to telenursing. In contrast, nurses with lower digital literacy, less engagement with online technologies, or no active presence on social media platforms may have been underrepresented in the sample. Second, the study employed a cross‐sectional design, which restricts the ability to draw causal inferences regarding the relationships between TAM–TPB constructs and intention to use telenursing. Longitudinal or experimental designs are needed to examine changes in attitudes and intentions over time and to better establish directionality. Third, all measures were based on self‐reported data, which may be subject to reporting bias and social desirability effects, even though anonymity was ensured. Participants may have overreported positive attitudes or intentions toward telenursing due to professional norms, perceived expectations, or desirability of technological competence. Furthermore, a significant limitation involves the measurement of prior training. This study relied on a simplistic assessment of whether participants had received training, without accounting for the quality, format, and duration of instruction. Differences between a brief introductory webinar and a comprehensive certification program could significantly impact a nurse's PEOU and behavioral intention, a nuance not captured in the current data. Only 34 participants reported prior telenursing training, limiting the precision of estimates and the generalizability of training‐related findings.

Finally, while preliminary analyses revealed no significant differences based on gender, the sample was predominantly female. This reflects the gender distribution of the nursing profession in Israel; therefore, caution should be exercised when generalizing the results to other healthcare systems.

Despite these limitations, our study offers valuable insights into the factors associated with nurses' intention to practice telenursing. By examining nurses' perceptions through an integrated TAM–TPB model, the model accounted for 74% of the variance in behavioral intention to use videoconferencing for telenursing. This proportion of explained variance was higher than that reported in previous studies using the TAM or TPB model separately.

## 5 Conclusions

This study highlights the value of a combined TAM–TPB framework for understanding nurses' perceptions and intentions to use videoconferencing for telenursing. Integrating both models provides a more comprehensive understanding of technology adoption among nurses, moving beyond the perceived usefulness emphasized by TAM to also consider the social and behavioral aspects highlighted by TPB.

Although prior telenursing training was not independently associated with intention after adjustment for the TAM and TPB constructs, structured training, together with a supportive organizational environment, may facilitate the implementation of telenursing and therefore warrants further evaluation. Future research should identify which training characteristics, such as duration, format, intensity, and practical components (e.g., brief webinars, simulation‐based learning, and structured certification programs), are most effective in supporting telenursing implementation through longitudinal and interventional study designs.

## Author Contributions

Study conceptualization/design: LS and SK. Data collection: SK. Data analysis: LS and SK. Study supervision: LS. Manuscript drafting and writing: LS and SK. Critical revisions for important intellectual content: LS.

## Conflicts of Interest

The authors declare that there are no conflicts of interest. All authors have contributed sufficiently to the project to be included as authors, and each meets the authorship criteria as defined by the International Committee of Medical Journal Editors. All authors have read and approved the final manuscript.

## Ethical Approval

The study was approved by the Ethics Committee for Nonclinical Studies at Bar‐Ilan University, Israel. The ethics form was signed by the committee head and the date of approval was November 17, 2022. The committee did not issue a formal ethics approval reference number. All procedures were performed in accordance with the relevant guidelines and regulations of the institutional ethics committee and the Declaration of Helsinki. Written informed consent was obtained from all participants digitally via the Qualtrics platform. At the commencement of the survey, participants were provided with a comprehensive explanation regarding the study's purpose, its anonymous nature, and the voluntary basis of participation. They were explicitly informed of their right to withdraw at any time without penalty. Participants were required to click a mandatory “I consent” button to proceed; those who did not provide consent or were under the age of 18 were automatically excluded from the study. No financial or material incentives were provided for participation.

This study did not involve patients or members of the public in the design, conduct, analysis, or preparation of the manuscript because the research focused solely on healthcare professionals' behavioral intentions regarding technology adoption.

## Funding

The authors have nothing to report.

## Supporting information



Supporting file 1: inr70263‐sup‐0001‐SuppMat.docx

Supporting file 2: inr70263‐sup‐0002‐Table1.docx

Supporting file 3: inr70263‐sup‐0003‐S2_correct_22.10.26.docx

## Data Availability

The data sets generated and/or analyzed during the current study are available from the corresponding author upon reasonable request.
