# Supplementary material for: Factors Associated With Nurses’ Acceptance of Videoconferencing in Telenursing: An Integrated TAM–TPB Analysis of Behavioral Determinants and Prior Training
Source: Int Nurs Rev. 2026 Nov 14;73(4):e70263. doi: 10.1111/inr.70263 (PMC13769146; doi:10.1111/inr.70263)
Supplement: Supplementary file 1 [file INR-73-0-s001.docx]

**Supplementary Figures**

**Supplementary Figure 1: Research Model and Hypotheses**

**Supplementary Figure 2 Confirmatory Factor Analysis (CFA) Model with Standardized Factor Loadings**

Confirmatory factor analysis (CFA) was conducted in AMOS 28 (Arbuckle, 1994) to test the hypothesized eight-factor structure of the questionnaires. Following recommendations for evaluating model fit, the model was assessed using five fit indices: the chi-square statistic $\left( \chi^{2} \right)$, the Root Mean Square Error of Approximation (RMSEA), the Normed Fit Index (NFI), the Comparative Fit Index (CFI), and the Tucker–Lewis Index (TLI). Consistent with commonly used guidelines (Hoyle, 1995), RMSEA values below .06 in combination with CFI, TLI, and NFI values above .95 indicate excellent fit, whereas RMSEA values below .08 and CFI, TLI, and NFI values above .90 indicate adequate fit.
Overall, the results supported the hypothesized eight-factor structure and indicated that the subscales are distinct. The chi-square statistic was significant, $\chi^{2}(406)=791.24,p<.001$. However, the chi-square test of model fit is known to be highly sensitive to sample size and tends to become significant in large samples (e.g., $n>200$), even when overall model fit is acceptable (Kyriazos, 2018). In the current sample, most alternative fit indices (apart from *NFI = .87*) indicated adequate fit (*RMSEA = .05; CFI = .93; TLI = .92*).

Arbuckle, J. L. (1994). Computer announcement amos: Analysis of moment structures. *Psychometrika*, *59*(1), 135-137.

Hoyle, R. H. (Ed.). (1995). *Structural equation modeling: Concepts, issues, and applications*. Sage.

Kyriazos, T. A. (2018). Applied psychometrics: sample size and sample power considerations in factor analysis (EFA, CFA) and SEM in general. *Psychology*, *9*(08), 2207.
