# Supplementary material for: Factors Associated With Nurses’ Acceptance of Videoconferencing in Telenursing: An Integrated TAM–TPB Analysis of Behavioral Determinants and Prior Training
Source: Int Nurs Rev. 2026 Nov 14;73(4):e70263. doi: 10.1111/inr.70263 (PMC13769146; doi:10.1111/inr.70263)
Supplement: Supplementary file 2 [file INR-73-0-s002.docx]

**Supplementary Table 1: Univariate analyses between TAM and TPB variables and**

**Telenursing past training**

|  | Telenursing past training | | | | t | p-value | Effect size |
| --- | --- | --- | --- | --- | --- | --- | --- |
|  | Yes  (n= 34) | | No  (n= 296) | |  |  |  |
|  | M | SD | M | SD |  |  | Cohen's d |
| **TAM** |  |  |  |  |  |  |  |
| Attitude towards use | 4.62 | 0.90 | 4.27 | 1.04 | 1.87 | 0.06 | 0.34 |
| Perceived usefulness | 4.25 | 0.99 | 4.07 | 1.00 | 1.02 | 0.31 | 0.18 |
| Perceived ease of use | 4.70 | 1.06 | 4.52 | 0.92 | 1.03 | 0.31 | 0.19 |
| **TPB** |  |  |  |  |  |  |  |
| Subjective norms | 4.37 | 0.97 | 3.66 | 1.06 | 3.73 | <.001 | 0.68 |
| Perceived behavioral control | 4.76 | 0.96 | 4.11 | 1.04 | 3.53 | <.001 | 0.64 |
| self-efficacy | 4.63 | 0.92 | 4.51 | 1.05 | 0.66 | 0.51 | 0.12 |
