# Supplementary material for: Factors Associated With Nurses’ Acceptance of Videoconferencing in Telenursing: An Integrated TAM–TPB Analysis of Behavioral Determinants and Prior Training
Source: Int Nurs Rev. 2026 Nov 14;73(4):e70263. doi: 10.1111/inr.70263 (PMC13769146; doi:10.1111/inr.70263)
Supplement: Supplementary file 3 [file INR-73-0-s003.docx]

**Supplementary Table S2: Sensitivity analysis: hierarchical regression predicting intention to use telenursing, adjusted for gender, education and seniority**

| **Predictor Variable** | **Step 1- Professional variables** | | | | **Step 2 +TAM** | | | | **Step 3 + TPB** | | | | | **Step 4 + Training &accessibility** | | | |
| --- | --- | --- | --- | --- | --- | --- | --- | --- | --- | --- | --- | --- | --- | --- | --- | --- | --- |
|  | **B*(se)*** | ***β*** | ***P*** | ***95%CI***  ***for* B** | **B*(se)*** | ***β*** | ***P*** | ***95%CI***  ***for* B** | **B*(se)*** | ***β*** | ***P*** | ***95%CI***  ***for* B** | **B*(se)*** | | ***β*** | ***P*** | ***95%CI***  ***for* B** |
| **Demographic/**  **Professional** |  |  |  |  |  |  |  |  |  |  |  |  |  | |  |  |  |
| Female | 0.100 (0.158) | .035 | .528 | [−0.211, 0.410] | 0.140 (0.094) | .049 | .139 | [−0.046, 0.325] | 0.087 (0.082) | .031 | .290 | [−0.075, 0.249] | 0.094 (0.083) | | .033 | .259 | [−0.069, 0.257] |
| Seniority | 0.013 (0.008) | .090 | .118 | [−0.003, 0.029] | 0.006 (0.005) | .043 | .217 | [−0.004, 0.016] | 0.001 (0.004) | .010 | .736 | [−0.007, 0.010] | 0.001 (0.004) | | .010 | .732 | [−0.007, 0.010] |
| Education | 0.065 (0.139) | .027 | .641 | [−0.209, 0.339] | −0.061 (0.083) | -.025 | .466 | [−0.224, 0.103] | 0.010 (0.072) | .004 | .893 | [−0.132, 0.152] | 0.007 (0.072) | | .003 | .923 | [−0.135, 0.149] |
| **TAM** |  |  |  |  |  |  |  |  |  |  |  |  |  | |  |  |  |
| Attitude towards use |  |  |  |  | 0.428 (0.063) | .370 | <.001 | [0.304, 0.553] | 0.304 (0.056) | .263 | <.001 | [0.193, 0.415] | 0.299 (0.057) | | .258 | <.001 | [0.187, 0.411] |
| Perceived usefulness |  |  |  |  | 0.387 (0.066) | .323 | <.001 | [0.258, 0.516] | 0.256 (0.059) | .214 | <.001 | [0.141, 0.371] | 0.257 (0.060) | | .215 | <.001 | [0.139, 0.374] |
| Perceived ease of use |  |  |  |  | 0.286 (0.051) | .225 | <.001 | [0.185, 0.386] | 0.091 (0.051) | .072 | .074 | [−0.009, 0.191] | 0.090 (0.052) | | .071 | .083 | [−0.012, 0.192] |
| **TPB** |  |  |  |  |  |  |  |  |  |  |  |  |  | |  |  |  |
| Subjective norms |  |  |  |  |  |  |  |  | 0.217 (0.047) | .195 | <.001 | [0.125, 0.309] | 0.211 (0.047) | | .190 | <.001 | [0.118, 0.304] |
| Perceived behavioral control |  |  |  |  |  |  |  |  | 0.310 (0.056) | .273 | <.001 | [0.199, 0.421] | 0.300 (0.057) | | .264 | <.001 | [0.187, 0.413] |
| Self-efficacy |  |  |  |  |  |  |  |  | 0.014 (0.044) | .012 | .755 | [−0.073, 0.101] | 0.010 (0.049) | | .009 | .837 | [−0.086, 0.106] |
| Telenursing past training |  |  |  |  |  |  |  |  |  |  |  |  | 0.104 (0.116) | | .027 | .370 | [−0.124, 0.333] |
| Accessibility to patient |  |  |  |  |  |  |  |  |  |  |  |  | 0.022 (0.052) | | .019 | .669 | [−0.081, 0.125] |
| **Model Statistics** | *R^2^ = .012, F(3,326) = 1.308, p = .272* | | | | *R^2^ = .654, F(6,323) = 101.857, p < .001* | | | | *R^2^ = .744, F(9,320) = 103.29, p< .001* | | | | | *R^2^ = .745, F(11,318) = 84.334, p< .001* | | | |
|  |  | | | | *ΔR^2^ = .642, F(3,323) =200.01, p < .001* | | | | *ΔR^2^ = .090, F(3,320) = 37.36, p < .001* | | | | | *ΔR^2^ = .001, F(2,318) = .495, p = .61* | | | |

**Note.** B = unstandardized regression coefficient; SE = standard error; β = standardized regression coefficient; CI = confidence interval are reported for the unstandardized regression coefficients (B); ΔR² = change in explained variance
